# Decision Processes about Condom Use among Shelter-Homeless LGBT Youth in Manhattan

**DOI:** 10.1155/2012/659853

**Published:** 2012-05-27

**Authors:** Geoffrey L. Ream, Kate F. Barnhart, Kevin V. Lotz

**Affiliations:** ^1^School of Social Work, Adelphi University, Garden City, NY 11530, USA; ^2^New Alternatives for Homeless LGBT Youth, New York, NY 10009, USA; ^3^Trinity Place Shelter, New York, NY 10025, USA

## Abstract

Health behavior interventions based on Theory of Planned Behavior address participants' personally-held beliefs, perceived social norms, and control over the behavior. New data are always needed to “member check” participants' decision processes and inform interventions. This qualitative study investigates decision processes around condom use among 81 homeless LGBT youth ages 18–26. Findings indicated considerable endorsement of the conventional policy of always using condoms, promulgated in HIV prevention education targeting this population. Although some participants reported risk behavior in contexts of sex work, survival sex, casual encounters, open relationships, and substance use, most were aware of these risks and consistently safe in those situations. Condoms use boundaries became vulnerable in states of emotional need and negative mood. The only effect participants acknowledged of homelessness on condom use was indirect, through negative mood states. The most prevalent context of condom non-use was with long-term primary partners, a potential area of vulnerability because, of 13 participants for HIV or HCV, nine mentioned how they had been infected, and all nine believed they had acquired it from a primary partner. Findings imply programs should emphasize HIV risk potential within long-term romantic partnerships and mental health services to remediate negative mood states.

## 1. Introduction

According to the theory of planned behavior [1], health behaviors like condom use [2] are influenced by personally-held beliefs and perceived social norms. In order to appropriately address the target population's shifting attitudes and values toward the target behavior, new data are always needed. This study concerns homeless lesbian, gay, bisexual, and transgender (LGBT) youth, a population with even higher rates of HIV sexual risk behaviors than heterosexual homeless youth [3–5]. Shelters and other programs serving homeless LGBT youth [6, 7] provide condoms and HIV education tailored to their specific needs. The content of this socialization depends on assumptions about their condom use beliefs and norms, e.g., that their elevated risk comes from greater involvement in survival sex [8] and substance use [9]. This study is a “member check” of those assumptions, open-endedly inquiring into homeless LGBT youths' decision processes in hopes of identifying risky beliefs and norms not already addressed.

 Experiences along the path toward homelessness help form the context of homeless LGBT youths' condom decision processes. For both LGBT and heterosexual youth, family conflict, violence, and/or abuse in the home environment are the most frequent causes of leaving home [10]. Parental substance abuse also contributes [11, 12]. Many enter the care of the child welfare system and, as counterintuitive as it seems that youth come to further harm in care [13], most LGBT youth in the child welfare system experience physical or sexual abuse and virtually all experience verbal harassment [6, 14–17]. About half of participants in a 3-city study of LGBT youth [18] reported having, at some point, sought the *relative safety* of the streets. LGBT youth in foster care also disproportionately experience multiple placements, which arguably normalizes living with strangers in serial relationships and diminishes their healthy sense of boundaries [19]. Experiences of sexual abuse at home or in foster care socialize them for sex work [20]. This background, along with home exposure to the use and sale of drugs [9], works toward preparing youth for “street careers” [11] and against preparing them for life in conventional society. Their progress toward conventional life goals is further obstructed by potential employers' discrimination based on gender-atypical self-presentation [7, 12]. Homeless LGBT youth also face unsafe conditions in shelters [6, 21], which forces them to remain “in survival mode” while ostensibly off the streets. The system tasked with extricating them from homelessness creates as many obstacles as opportunities, causing some youth to stop trusting intervention efforts and believe that their place is on the streets [22].

 The best-known contributors to HIV risk behaviors among homeless LGBT youth are sex work/survival sex and substance use [23]. Sex work customers generally pay more for unprotected sex [24], and sex workers themselves may be willing to compromise about condoms when in great economic need [25, 26]. Transgender youth are at particular risk for sex work involvement [27]. Sex work and having friends involved in sex work are factors contributing to higher rates of sexual victimization among homeless LGBT youth [28]. Although survival sex and hard drug use are correlated among homeless youth [5, 29], it does not follow that the most prevalent route to HIV risk among most of them is survival sex to support a hard drug habit, as only a minority have any experience at all with hard drugs [9, 23]. It is also questionable whether the association between HIV risk and substance use is because substances' effects lead to lapses in judgment in sexual encounters because some substance use with sexual activity is intentional, to enhance the experience and reduce inhibitions [30, 31]. There has been a general call for empirical research to address unexamined assumptions about the mechanism of effect between substance use and HIV risk behavior [32].

 Other factors influencing homeless LGBT youths' condom use decisions are emotional, including depressed mood [33–35] and issues surrounding long-term romantic relationships. Our literature search did not find any studies specifically about condom use in homeless LGBT-identified youths' romantic relationships, but the general pattern among young people is to use condoms with casual partners and not use condoms with intimate long-term partners [36]. Non-use of condoms is also associated with fear of losing a long-term relationship [37], and insistence on condoms in a long-term relationship may cause partners to suspect infidelity [38, 39]. In sex work, which is part of homeless youths' milieu even if they do not participate [22], it is normative to forego condoms with long-term partners in order to distinguish long-term partners from sex work clients [40–43]. It follows, therefore, that long-term relationships should be assessed as potentially part of the risk context for homeless LGBT youth.

 Shelters and other programs for homeless LGBT youth often provide HIV prevention education, employing a rationale based significantly on theory of planned behavior (TPB; [1]) as applied to condom use [2]. TPB specifies that a health behavior is more likely when people judge the behavior to be acceptable in the eyes of significant others, believe the behavior will produce positive outcomes, and perceive that they have control over the behavior. Socialization messages of organizations serving homeless LGBT youth, therefore, affirm the efficacy of condoms in preventing HIV, contravene known myths about condoms, promote HIV prevention as an LGBT community issue, and educate youth about effective condom use and negotiation skills, delivering a message that is consistent with a comprehensive sex education approach [44, 45]. Organizations serving homeless LGBT youth aim to increase both perceived and actual control over condom use through providing free condoms and, under the premise that youth whose basic needs for food and shelter are met will have less economic incentive to engage in HIV risk behavior [46], they also provide shelter, case management, and several other supports. In addition to these efforts targeting control, organizations serving homeless LGBT youth also provide education and socialization targeting attitudes and beliefs about condoms. Because new myths, norms, and vulnerabilities always arise, new information must be continually gathered and integrated into interventions.

Qualitative methods are suitable for exploring the content of beliefs and attitudes [26, 47]. This study was, therefore, qualitative, and explored the following research questions: (1) on what basis do shelter-homeless LGBT youth in Manhattan choose to use condoms? (2) How do risk contexts of homelessness, sex work, psychological stress, and norms like open relationships affect their thought processes about condoms?

## 2. Methods

### 2.1. Participants and Recruitment

 Eighty-one participants were recruited from two shelters for homeless and street-involved LGBT youth in Manhattan. The shelters' target populations were youth 18–24. One shelter also allowed existing clients who had “aged out” to return for case management and drop-in services; such clients, were generally housing insecure, did not have a realistic goal of independent living and were in the process of transitioning to long-term adult supportive housing. The age range of participants was, therefore, 18–26. All participants were sexual minorities [48] in that they identified as LGBT and were also attracted to, dating, and/or having sex with people of their own biological sex—these shelters had a policy of referring youth who were not sexual minorities to other programs. All participants either resided in the shelters or close enough to travel there to receive case management and/or drop-in services. Consistent with community-based research principles, the interviewers had built a relationship with these programs for over a year through volunteering, pro-bono consulting, and other contributions. The protocol was reviewed and approved by the IRB at the principal investigator's institution. At the beginning of data collection, recruitment posters were placed conspicuously in program facilities, offering $10 public transportation fare cards for participation. The response was immediate and enthusiastic. As this initial response diminished in momentum, sampling became more theoretical [49, 50] as participants were invited to balance the sample for race, gender (including transgender) and sexual orientation categories. HIV-positive participants and those involved in sex work were oversampled in order to have sufficient representation in analyses.

### 2.2. Interview Protocol

 The principal investigator (a social work professor and researcher) and a trained research assistant conducted all interviews. After initially agreeing to participate, interviewers sat with participants in private offices within program spaces and read a scripted explanation of informed consent. After participants asked any questions they had and verbally consented to be in the study, interviewers began digital recording and again asked for verbal consent to record the interview. To preserve confidentiality and comfort, signed consent forms were not used. Interviews began with basic demographics and housing history. The condom-related questions began simply with, “When you have sex, how do you decide whether to use a condom?” and continued with prompts about specific risk contexts suggested by earlier research, for example, sex work, survival sex, trust in a long-term partner, open relationships, and drugs/alcohol. Participants were then asked to recall a specific decision about condom use and describe their thought process about it. They were also asked for their HIV status if they had not disclosed it already. Finally, they were asked if there was any message they wanted to communicate to service providers. Duration of interviews ranged from roughly 30 to 90 minutes. The same interview protocol was used for all participants, with additional follow-up questions asked of participants with stories to tell with respect to specific themes of interest, for example, sex work. The principal investigator and research assistant transcribed all interviews. Interviews were the only source of systematic data for this study.

### 2.3. Data Management and Analysis

 Interview audio files were moved off of digital video recorders as soon as possible and, when not being transcribed or analyzed, stored in an encrypted directory on an external hard drive in a locked university faculty office. The principal investigator and a research assistant applied thematic analysis [51] to the data. This involved drawing initial codes from interview transcripts and collecting them into themes. Themes were then applied to the entire data set, refined as appropriate, and assessed for both prevalence (i.e., how many respondents mentioned them) and how “key” they were to the overall story the data were trying to tell. Consultation about the coding process was obtained from two researchers experienced with ethnography of high-risk subcultures, and emerging themes were compared with insights of workers and research team members who were in regular direct contact with the youth as themes were identified. Data analysis began soon after the first few interviews were collected, and the interview protocol was refined in response to emergent themes in an iterative process. Data collection began to wind down after saturation was reached, with some additional interviews conducted so that all shelter clients who desired to participate could be included. Several themes turned out to be exclusively about housing history, and those are not reported here. All of the major themes about condom decision processes are reported below.

## 3. Results

### 3.1. Participant Characteristics

Participants ranged in age from 18–26 years and most lived in an emergency shelter or transitional living program (TLP). Participants older than 24 were generally former shelter clients returning for case management and drop-in services. Most of these clients lived in adult emergency shelters or long-term adult supportive housing like the single-room occupancy accommodations provided by the HIV/AIDS Services Administration or Department of Homeless Services. In terms of gender, 61% of participants were male, 21% male-to-female transgender, 16% female, and 3% female-to-male transgender. Racially, 12% were white, 52% Black, 30% Latino, 3% Native American, and the rest of other or mixed race. All were attracted to, and/or primarily had sex with, people of their own biological sex, including the “trans-amorous” males who preferred male-to-female transgender partners. The vast majority of participants had been tested for HIV recently enough to be relatively confident of their status. Only two had never been tested. HIV-positive or HCV-positive status was reported by 16% of participants. At least one occasion of sex work was reported by 37%. Involvement in sex work did not seem to be specific to any race, gender, or sexual identity.

 In the presentation of results below, figures for age have a random number between −1 and 1 added to them in order to preserve anonymity. Also to preserve anonymity, we labeled every response from someone positive for HIV or Hepatitis C with “Pos” and deliberately did not clarify which infection the participant had.

### 3.2. “Wrap It Up” (Always Use Condoms)

Condom-related socialization in LGBT youth services favors simply using a condom every time and maintaining rigid boundaries against unsafe sex. Statements in 31 interviews reflected this mentality, named by seven interviewees as “wrap it up” or “strap up.” Components of this mentality included the following.

- Insistence on condoms, even if it meant not having sex:

*If you don't want to use condoms, we not um gonna do nothin' (Latino gay male, 23).*

(ii) Using condoms even if aroused, high, drunk, tired, or averse/allergic to latex:

*She says um, baby, where's my covers at? I'm so drunk that I'm like my eyes is so closed so I'm like, “why would it…why in the hell would I know where your covers is at?”…we wound up having sex that night…With a condom. (Black trans-amorous male, 23).*

(iii) Associating condoms with social responsibility and conventionality (in the following quote, note “coward” as a moral judgment upon someone who would put a partner at risk):

*He was like, I love you, and as soon as he said that, I just I got off him, and I made him put a condom on. Because I don't want to be a coward…I couldn't be that cruel (Latino gay male, 25, Pos).*

(iv) Repudiation of specific beliefs common among peers, particularly the idea that unsafe sex is okay with a partner that has proven HIV-status or that one “trusts” on other levels:

*She can show me all her papers and be clean and I'll still use it (Latino bisexual female, 19)*

*The best looking person can have it (Black bisexual male, 20, Pos).*

*I don't care like how long we've been together we can be together for week two weeks two years and the whole using condom thing will never change (White trans-woman, 21).*

*Maybe you trust them and they don't even know that they have something (White/Latino gay male, 18).*

Also emergent under this theme was a quantitative finding: of the nine HIV-positive participants who mentioned how they had become infected, *all* believed they had acquired it from primary partners.

### 3.3. Trust in a Long-Term Partner Not to Cheat or Be HIV-Positive

 Although the most *conventional* policy was “wrap it up,” the most *common* policy, cited in 49 interviews, was one of calculated risks based on trust in long-term partners not to put them at risk for an STI. HIV-positive and HCV-positive participants also engaged in calculated risk of superinfection or additional STIs. This policy often included benchmarks for how long they would have to be together to forego condoms, for example, two years (Black gay male, 20), or 2-3 months (Black lesbian, 20). Some also observed a policy of getting tested together, mentioned in 17 interviews:

*If …we both had been tested and then really believed that they were safe and if we'd been together for two years or married or something…maybe (Black trans-man, 24).*

HIV- or HCV-positive participants could avoid superinfection from unprotected sex with primary partners through serosorting and trusting partners to be faithful. This was reported in 2 interviews:

*We have sex without (condoms). I know we shouldn't, but I know his status and I know my status, I know I caught mine from him, so we have sex every night without a condom (Latino gay male, 22, Pos).*

In 3 cases, associating non-use of condoms with trust led to an inference that a long-term partner who suddenly insisted on condoms was cheating:

*We had a fight actually, our first fight after a whole year. He accused me of sleeping with somebody else because I wanted him to use a condom…He was like y'all trust me you don't trust me…just has his ways of making you feel so bad for him and then I'm like you know what just to prove I trust you I won't use a condom. That's where I fucked up (Black gay male, 24).*

### 3.4. Love and Emotional Intimacy with Long-Term Partners

In 17 interviews, unprotected sex with long-term partners was motivated by, or an indicator of, emotional intimacy. These emotion-based choices are distinct from the rational, calculated risk of those made under the “trust” norm:

*When I loved him, I trust him. I had, I mean, I felt like, he was like the same way I was, and I wasn't messing around, I wasn't lying, I know that (Black trans-woman, 25).*

### 3.5. “The Moment” and Sexual Pleasure

Willingness to compromise safety for pleasure was mentioned in 9 interviews. Such choices included not insisting on condoms, not disclosing HIV-positive status, not taking “using a condom during oral sex seriously” (Black gay male, 23), or dropping the condom requirement in a relationship after days instead of the months it would take to be sure of a partner's HIV status. In contrast to the trust norm and affect heuristic, these participants actually factor considerations like discomfort using condoms into cost-benefit analyses around HIV risk:

*Condoms irritate me for some reason I…it might be a mind thing, but in my mind, I think they irritate me, they hurt, I don't like the way they feel, they're uncomfortable…And, um, he didn't really like to use them anyways, he liked to have raw sex like I did so it was…a two-way thing. We just stopped using them at the end (Latino gay male, 23, Pos).*

Beliefs and experiences in which the moment was clearly more important than safety were mentioned in 13 interviews. Their motivation could be sexual desire, emotional vulnerability, or both:

*Because sometimes, I mean, there's been sex, there's been sex everywhere. In clubs, in cars, in the streets, in the alleys and parks in trains and bathrooms. You name it, I've done it…heat of the moment, and you're drunk or you're high, you're not thinking about consequences, you're just thinking about getting it. Some people have brought it up, or I've brought it up, and I have just like, “don't worry I'm safe.” And that's like one of the sexiest things you can say; that kind of ruins the whole thing if you say “I don't have HIV” or “I don't have no STDs,” it's just stop, just a minute, it's just sexier to say “I'm safe, don't worry, I'm safe” (Black gay male, 22).*

### 3.6. “Go with the Flow” to Avoid Awkwardness of Condom Negotiation

Yet another policy, evident in 10 interviews, was to avoid the awkwardness of condom negotiation and simply “go with the flow.” It is distinct from the above in that the mindset in these encounters is not participants' own satisfaction but desire to please, or avoid problems with, either romantic or sex work partners:

*I knew if I had said something about it, it would set up a drama (Black trans-woman, 23, Pos).*

*It all depends on my customer…if he asks me first, then I say okay. If he don't ask me, then I'm not going to use it…(Interviewer: So you pretty much go with…) the flow (White trans-woman, 20).*

### 3.7. Risk Context: Open Relationships

Engagement in relationships without the expectation of exclusivity was reported in 23 interviews. (Some participants initially interpreted “open relationship” to mean one that was not secret or closeted; this was clarified for them.) When asked about harm reduction rules, 12 reported using condoms with both their primary partner and other partners:

*With me, it was mandatory to use condoms every time. It was an open relationship, I knew at the end of the day that the other person was going to have sex with somebody else…For you to touch me, you know, and feel that you didn't have to be protected, I just, to me, that doesn't sit right with me, I don't feel right. I feel like the best thing is to protect yourself (Black lesbian, 20).*

Eight reported using condoms with other partners, but no condoms with their primary partner:

*And, when I was with him, I was with him, when he was with me he was with me, but when we're separated, we're single, and we can do whatever the fuck we wanted, as long as we used a condom and we didn't bring something back (Latino gay male, 24, Pos).*

Three described situations when the nature of the relationship changed, so did the rules about condoms:

*He was like, “you can have sex with this person, I can have sex with that person, is that all right with you? Can we do it like that?” I was like, “sure,” ‘cause I really didn't care about him like that…then…I wouldn't do it with him ‘cause I'm in love with him, he was like, “fine, we won't do it” (Native American gay male, 21, Pos).*

*Those agreements actually didn't last because my partner didn't meet with them…He didn't want to use condoms (Black gay male, 20).*

### 3.8. Risk Context: Sex Work

Homeless LGBT youth often encounter opportunities to trade sex for money or housing, even though only a minority (at least, within this shelter-homeless population) actually participate. Their overall norms for condom use—that is, that protection should be involved in encounters with partners they do not love, trust, desire, or plan to be with long term—generalize to protect them in sex work situations. Twenty-five interviews of youth who had engaged in sex work affirmed adherence to this norm:

*I hate it when like the client's like “I don't have anything, I don't have anything.” I'm like, “I'm not going to take that chance, I'm sorry. I don't know you that well, I just met you right now, and you think I'm going to believe you?” (Black lesbian, 18).*

Seven interviews of youth who had engaged in sex work reflected willingness to not to use condoms in a sex work context:

*I needed more money, therefore, I asked him “I can take this condom off if you give me a extra…extra.” And they usually say “Well, how much,” and they throw me a $20. If they throw me anything less than a $20, I say “no, I can't do it” (Black trans-woman, 21).*

Even for youth who do not engage in sex work, the presence and availability of sex work is still part of their environment. Seven interviews of participants who had never engaged in sex work reported receiving and declining offers:

*I helped bring her bags in her car, I was 17 years old she was about 29 or 30 she said she'd pay me $500 to lick my ass. And that was shocking to me. She had the money too. And I'm not going to lie, I wanted the money bad but I couldn't bring myself to do it. I've had so many offers. So many offers (Black gay male, 21).*

An additional three mentioned no actual sex work or offers for it, but did have “gray area” experiences between casual sex and sex work:

*Gets $200 from the ATM, hands me $180…catching a cab back…I'm finger poppin' her and shit, we having sex…when we got to the place, she hops out of the cab and runs straight across the street …yelling, “ Oh, all you want me for is sex!”… I don't even know you from a hole in the wall, but we still fucked. And she just runs…the guy at the front desk was like, “Oh, she always does this” (Black trans-amorous male, 22).*

*I fucked him with a condom and I woke up and there was like $300 right beside me. So I don't know if I was paid I was a paid ho; I probably was. I'm trying not to think of it like that (Black bisexual female, 24).*

An additional seven interviews that received none of the previous four codes still reported peer encouragement toward sex work:

*A lot of people of offered me like, you should put up an ad on Craig's List…there are many a time, because a lot of my friends, a lot of them, do sex work. And many a time, the money that they bring in, sometimes that thought is like a pounding thought in my head (Black trans-amorous male, 20).*

### 3.9. Risk Context: Negative Emotional States

Psychological stressors such as low self-esteem, depression, grief, and a general loss of meaning and purpose were described as wearing down condom boundaries in 23 interviews. These situations are distinct from the cost-benefit analyses mentioned above. Rather, psychological stress diminished these youths' capacity to enforce boundaries:

*I was in love. And, it was kind of like my heart was broken, depression fell in, and I just didn't give fuck, if it happened… I remember a point in time where …I actually wanted to get HIV…I figured if I get it, I'd die. If I died, then, no one could say it was suicide…I was trying to destruct myself without having to be the weapon (Latino gay male, 24, Pos).*

Only one interview affirmed, while 55 repudiated, that housing status had any *direct* effect on condom use. Any effect of homelessness on condom use was perceived to be *indirect*, through negative emotional states:

*I think that plays a part in me not caring when I engaged in sex, because I didn't care about myself. I'm just now getting to a place where I'm starting to care more about me. You know, ‘cause, back then, I didn't care, you know, I was still dealing with a lot of hurt and a lot of pain from my past, and growing up and the different experiences with being gay and everything like that. That, it made my self-worth seem like it was, I had none. So, I think that when a person is homeless, you're still dealing with depression, which comes from being homeless, or it could come from the experiences in their lives (Black gay male, 25).*

This effect of negative emotional states could intersect with the norm of unprotected sex with primary loved partners in order to create a particular risk context:

*When you're homeless and you have nobody you just want to feel like you've got somebody and you, when it comes to sex, you, I think that not using a condom expresses that you want, um, you want more from the person or you want them to realize that anything between you is greater than using a condom (Black gay male, 21, Pos).*

### 3.10. Risk Context: Drugs and Alcohol

 Although current drugs of choice in this sample were primarily marijuana and alcohol, hard drugs were still a part of their risk context. In 14 interviews, participants reported that they never use drugs or alcohol. An additional 12 reported that they do not use drugs or alcohol to the point of intoxication. Another 12 reported, with various degrees of intentionality, not having sex while high or drunk:

*I've never really tried to have sex under the influence, never really wanted to do that. I have gotten drunk after sex of course, because of the depression that I feel from it (Bisexual black male, 22).*

And 11 reported that, although they would have sex while high or drunk, it either had no effect on their condom use or, because they were fully aware of the dangers, made them more careful:

*Oh hell, no…I always make sure that I have a condom…doesn't matter how drunk I am…doesn't matter how drunk he is. It's always a condom (Latino gay male, 20).*

*Alcohol, it impairs your judgment, so you always have to be on point. Alcohol, it will fuck you up. (Interviewer: Marijuana?) Please. That shit don't do nothin' (Black lesbian, 21).*

Some experience with hard drugs was reflected in 16 interviews. Only two reported using sex work or survival sex to support a habit:

*Yes, I have had sex before for money… to keep my (cocaine) habit going…I was like 14, 15, and I would go and sell my body and then go back home. And my parents thought I had a job (Bisexual black male, 22).*

### 3.11. Transformative Confrontation with HIV and HCV

HIV “scare” experiences (few participants were more than distantly aware of HCV) were reported in 18 interviews. These included seeing the effects of HIV and HCV on others, catching other STIs themselves, or realizing, after an unprotected encounter, that they had put themselves at risk. This usually made them revise their condom policies:

*That syphilis shot is no joke that's some big ass needles …after this whole syphilis incidence, I was not playing. So I don't care how long you're with me, you're going to use a condom or whatever until I feel comfortable. Actually, you might have to use a condom forever, because I'm going to be mad paranoid now, I'm going to be like super paranoid, I'm going to cover myself in Saran wrap…I might catch something that you won't have a shot for (Black gay male, 24).*

Among the 13 youth who were positive for HIV or HCV, nine reported that their infection was a turning point in their lives:

*I just found out like three months ago…it felt like you're invincible and then all of a sudden you're infected and everything changes. Your demeanor, the way that you carry yourself, your whole life, it's….it's all different, and it doesn't seem like it's a positive thing, ‘cause truthfully it really isn't. But, it all depends how you deal with it… I've really done a lot of support groups ‘cause that was really what kind of supported me along the way to stay positive…having someone to talk to that's been through it before (Latino gay male, 19).*

## 4. Discussion

The purpose of this investigation was to “member check” homeless LGBT youths' condom-related attitudes and beliefs within the context of homeless LGBT youth services' tailored safer sex socialization. Findings suggest that the message is getting through: most participants knew that the “correct” answer to our initial question about their decision process about condoms was “always use condoms,” and very few of their responses throughout these interviews reflected misinformation about condoms [52]. However, these participants also employed situational ethics. They were very aware of the risks associated with survival sex, sex work, casual encounters, and substance use. Some acknowledged various degrees of risk behavior, but most reported being strictly safe in those situations. Although open relationships in which partners trust each other not to “bring anything back” are arguably a gray area of safety, most participants who were in open relationships remained safe by insisting on condoms with primary partners as well. The factor that seemed reliably associated with lower vigilance was emotional decision making about condoms, e.g., enhancing pleasure in the moment, avoiding awkwardness around condom negotiation, being in love, or experiencing negative emotional states that weakened boundaries and heightened emotional need. In fact, to the extent that homelessness was acknowledged to have any effect on condom use at all, it was perceived as indirect, through negative emotional states.

Some participants made less-safe choices about condoms by factoring reduced sexual pleasure, physical discomfort with condoms, and awkwardness of condom negotiation into their cost-benefit analyses. Because HIV prevention education already addresses these condom non-use motivations [45], those findings are not as meaningful toward our goal of identifying risk beliefs and norms that are under-emphasized in programs' condom socialization messages. Rather, the situational ethic of condom use that we found to be both the most numerically prevalent and the least emphasized in existing condom socialization messages was trust in a long-term partner to be faithful and HIV negative. This trust ethic was practiced according to the norm of non-use of condoms as a way of both expressing trust and intimacy with primary partners and of distinguishing primary partners from casual partners. Organizations involved in this study have already adjusted their safer sex socialization messages in order to address that norm. They were particularly motivated to do so in light of our discovery that, of 13 HIV+ or HCV+ youth who participated in these interviews, all nine who mentioned how they had become infected believed they had acquired it from a primary partner. These organizations were further concerned because, in case management, clients report that couples' housing is far more comfortable and stable than emergency shelters, which suggests that the system subtly shifts youths' cost-benefit analyses toward forming and staying in long-term romantic relationships. Given the connection between condom use and stability/intimacy of long-term romantic relationships [36], this may create a cognitive dissonance process in which youth reevaluate how much they trust their partners.

These findings can be usefully applied according to TPB principles of attitude, belief, and control [1, 2]. Control over condom use appears to be firm within this population, except in cases of emotional vulnerability. Few responses from these shelter-homeless youth reflected non-use of condoms for drug-related or economic factors, which are probably more characteristic of the experiences of street-homeless youth [53]. However, the attitude connecting non-use of condoms with long-term relationships, coupled with the belief that this is an effective harm reduction strategy, is an apparent area of vulnerability. Unless HIV education programs address situations specifically faced by their clients, clients make conjectures about the risks associated with their behavior and usually underestimate it [54]. Findings from this study support a recommendation that HIV programming for homeless LGBT youth should address long-term relationships as a potential risk context. Programs serving homeless LGBT youth should also strive to provide counseling and mental health interventions to address negative emotional states, which youth identify as a more proximal risk factor for HIV risk behavior than homelessness itself.

One limitation of this study is that, although qualitative interviews were arguably the most appropriate method to address the research questions, the study had to rely exclusively on that method. This was mainly because limited resources did not allow for additional study components like street-based field observations that would have allowed us to triangulate and contextualize data. The team was also, having been immersed in the shelter service context for years before the study began, arguably too “native” to make field observations of the shelters themselves. Because full life-history interviews would not have been practical due to the exigencies of the study setting, the scope of the interviews also had to be narrowly focused on condom decision processes and the context of homelessness. Our results also cannot be expected to generalize to actively using problem hard drug or alcohol users (a behaviorally and demographically distinct population—see Hickler and Auerswald [29]—which shelters usually cannot accommodate), youth living on the street, or beyond the NYC context. This study's primary strength was adherence to a community-based research methodology of building long-term relationships with the organizations and their clients. Youth and workers authentically trusted researchers. We believe this factor, more than any other, contributed to youths' willingness to go beyond giving the “right answers” according to the programs' messages and authentically describe the complexity and details of their condom decision processes.
